# Casein kinase 2 modulates the spindle assembly checkpoint to orchestrate porcine oocyte meiotic progression

**DOI:** 10.1186/s40104-020-00438-1

**Published:** 2020-04-08

**Authors:** Xiayan ShiYang, Yilong Miao, Zhaokang Cui, Yajuan Lu, Changyin Zhou, Yu Zhang, Bo Xiong

**Affiliations:** College of Animal Science and Technology, Nanjing Agricultural University, Nanjing, 210095 China

**Keywords:** CK2, CX-4945, DNA damage, Meiotic maturation, Porcine oocytes, Spindle assembly

## Abstract

**Background:**

CK2 (casein kinase 2) is a serine/threonine-selective protein kinase that has been involved in a variety of cellular processes such as DNA repair, cell cycle control and circadian rhythm regulation. However, its functional roles in oocyte meiosis have not been fully determined.

**Results:**

We report that CK2 is essential for porcine oocyte meiotic maturation by regulating spindle assembly checkpoint (SAC). Immunostaining and immunoblotting analysis showed that CK2 was constantly expressed and located on the chromosomes during the entire oocyte meiotic maturation. Inhibition of CK2 activity by its selective inhibitor CX-4945 impaired the first polar body extrusion and arrested oocytes at M I stage, accompanied by the presence of BubR1 at kinetochores, indicative of activated SAC. In addition, we found that spindle/chromosome structure was disrupted in CK2-inhibited oocytes due to the weakened microtubule stability, which is a major cause resulting in the activation of SAC. Last, we found that the level DNA damage as assessed by γH2A.X staining was considerably elevated when CK2 was inhibited, suggesting that DNA damage might be another critical factor leading to the SAC activation and meiotic failure of oocytes.

**Conclusions:**

Our findings demonstrate that CK2 promotes the porcine oocyte maturation by ensuring normal spindle assembly and DNA damage repair.

## Introduction

CK2 (casein kinase 2) is an ubiquitously expressed and highly conserved serine/threonine protein kinase that forms a tetramer containing two catalytic (α and/or α´) subunits and two regulatory β subunits [1]. According to the specific functions, its catalytic and regulatory subunits may form αα´β2, α2β2, α´2β2, or unassembled molecules [2, 3]. CK2 is responsible for the phosphorylation of many substrates in various pathways within a cell, using ATP or GTP as the phosphate source [4]. It has been reported to CK2 participates in cell cycle regulation [5], cell proliferation, DNA damage repair [6, 7], apoptosis [8, 9], and other cellular processes [4, 10, 11]. Notably, CK2 is overexpressed in cancer cells of the prostate, colon, lung and breast, and takes a critical part in cancer progression by regulating a number of signaling pathways, including PI3K/Akt, Wnt/β-catenin and MAPK [12–14]. The anti-apoptotic role of CK2 allows the cancerous cells to escape cell death and continue proliferation, thus becoming an effective drug target for cancer treatment [15, 16]. CX-4945, as a first oral bioavailable CK2 small molecule inhibitor, exerts anti-proliferative activity in human cancer cells by inhibiting cell cycle and PI3K/Akt signaling pathway [17].

In addition, studies have reported that CK2 is required for individual development in mammals. Deletion of the catalytic subunit CK2α’ in mice results in the male infertility by exhibiting the nuclear abnormalities and apoptosis in the sperm [18, 19]. Whereas, deletion of CK2α in mice leads to the death of mid-gestation, which is caused by the defects in heart and neural tube, revealing the specific function of CK2α in these organ development [20]. Besides, mice lacking of the regulatory subunit CK2β displays the early embryonic lethality in a cell autonomous fashion [21]. The embryos of CK2β knockout mice are dead shortly after implantation and show reduced cell proliferation without signs of apoptosis [21]. Moreover, CK2β is essential for the female fertility and ablation of CK2β causes the follicle atresia and premature ovarian failure through suppressing PI3K/AKT and DNA damage response signaling pathways [12]. CK2β directly binds and inhibits Mos to prevent premature MAPK activation and oocyte maturation [22]. Although increasing studies have demonstrated the essential functions of CK2 in the various biological processes, its exact role in mammalian oocyte meiosis remains largely unknown.

In the current study, we explored the functional roles of CK2 on the porcine oocyte meiotic maturation. We examined the localization and expression patterns of CK2 in the various developmental stages of meiotic progression. We also employed the CK2-specific inhibitor CX-4945 to validate the effect of CK2 activity on the polar body extrusion, spindle assembly, microtubule stability and DNA damage level in porcine oocytes.

## Methods

### Antibodies

Rabbit polyclonal anti-CK2 antibody, rabbit polyclonal anti-Gapdh antibody, and rabbit monoclonal anti-γH2A.X antibody were purchased from Cell Signaling Technology (Danvers, MA, USA); Mouse monoclonal anti-α-tubulin-FITC antibody and anti-acetyl-α-tubulin (K40) antibody were purchased from Sigma (St. Louis, MO, USA); Rabbit polyclonal anti-phosphoCK2 and sheep polyclonal anti-BubR1 antibodies were obtained from Abcam (Cambridge, MA, USA); goat anti-mouse IgG (H + L)-FITC, goat anti-rabbit IgG (H + L)-FITC and donkey anti-sheep IgG (H + L)-FITC were obtained from Zhongshan Golden Bridge Biotechnology (Beijing, China).

### Collection and *in vitro* maturation of COCs

COCs (cumulus-oocyte complexes) with a compact cumulus mass were collected from medium-sized follicles of porcine ovaries using the disposable syringe, and then transferred to the *in vitro* maturation medium (improved TCM-199 supplemented with 0.5 μg/mL FSH (follicle-stimulating hormone; Ningbo Second Hormone Factory), 0.5 μg/mL LH (luteinizing hormone; Ningbo Second Hormone Factory, Zhejiang, China), 0.57 mmol/L cysteine (Sigma), 10 ng/mL EGF (epidermal growth factor; Sigma), 50 μg/mL streptomycin and 75 μg/mL penicillin). A group of 80 COCs was cultured in 500 μL of maturation medium covered with 200 μL paraffin oil to the specific developmental stages for the subsequent analysis at 38.5 °C in a humidified atmosphere of 5% CO_2_.

### CX-4945 treatment

CX-4945 (Selleck, Munich, Germany) was dissolved in DMSO and then diluted with the culture medium to a working concentration of 1, 5, 10 or 20 μmol/L, respectively. The final concentration of DMSO was not more than 0.1% in the culture medium. Oocytes at GV, GVBD, M I and M II stages were collected following the culture in the maturation medium containing CX-4945 for 0 h, 20 h, 28 h and 44 h, respectively.

### Immunofluorescence and measurement of fluorescence intensity

DOs (denuded oocytes) were fixed in 4% PFA (paraformaldehyde) for 1 h at RT (room temperature), and then transferred to the solution (20 mmol/L HEPES, pH 7.4, 1% Triton X-100, 50 mmol/L NaCl, 3 mmol/L MgCl_2_, 300 mmol/L sucrose in PBS) for permeabilization overnight. After incubation in blocking buffer (3% BSA/PBS) for 1 h at RT, oocytes were stained with α-tubulin-FITC antibody (1:200), acetylated-α-tubulin antibody (1:100), γH2A.X antibody (1:200) or BubR1 antibody (l:50) at 4 °C overnight, and then incubated with the appropriate secondary antibodies for 1 h, followed by counterstaining with PI (propidium iodide) or Hoechst 33342 for 10 min at RT. Lastly, oocytes were mounted on the glass slides for acquisition of images using the laser-scanning confocal microscope (Zeiss LSM 700 META confocal system).

The images from both control and CK2-inhibited oocytes were obtained by following the same immunofluorescence procedure and parameter setups of confocal microscope. Then, the fluorescence intensity of region of interest in the images was measured using Image J (NIH, USA).

### Immunoblotting analysis

50–100 porcine oocytes were lysed in 4 × NuPAGE™ LDS sample buffer (ThermoFisher, USA) containing protease inhibitor. Proteins were separated by SDS-PAGE and then transferred to PVDF membranes. Membranes were blocked in TBS containing 0.1% Tween 20 and 5% low fat dry milk for 1 h and then incubated overnight at 4 °C with anti-CK2 antibody (1:1,000), anti-acetylated-α-tubulin antibody (1:1,000) or anti-Gapdh antibody (1:5,000). After several times of washes in TBS containing 0.1% Tween 20 and incubation with HRP-conjugated secondary antibodies, the protein bands were developed with ECL Plus (GE Healthcare, USA) and acquired by Tanon-3900 imaging system (Tanon, China).

### Statistical analysis

Data were expressed as mean percentage (mean ± SEM) of at least three independent replicates. Differences between two groups were analyzed by Student’s *t* test. Multiple comparisons between more than two groups were analyzed by one-way ANOVA test using SPSS16.0 statistical software (IBM, USA). *P* < 0.05 was accepted to be significant.

## Results

### CK2 localizes to chromosomes in porcine oocyte meiosis

**Fig. 1:**
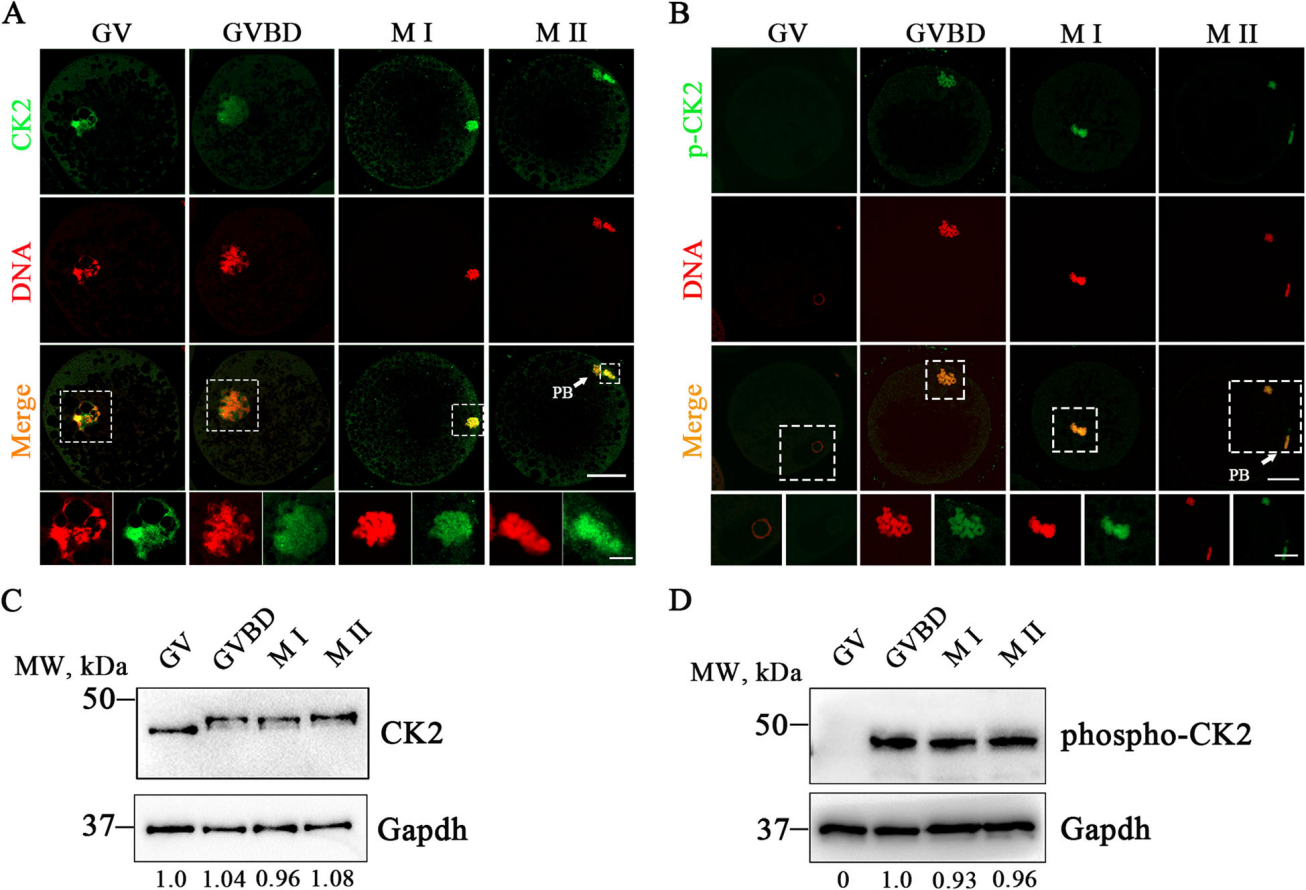
Subcellular localization and protein expression of CK2 during porcine oocytes meiotic maturation. **a** Representative images of CK2 localization during oocyte meiosis. Porcine oocytes at GV, GVBD, M I and M II stages were immnunostained with anti-CK2 antibody (green) and counterstained with PI (propidium iodide, red). Scale bar, 30 μm; 5 μm. **b** Representative images of phosphorylated CK2 localization during oocyte meiosis. Porcine oocytes at GV, GVBD, M I and M II stages were immnunostained with anti-phosphoCK2 antibody (green) and counterstained with PI (propidium iodide, red). Scale bar, 30 μm; 5 μm. **c** Protein levels of CK2 at specific time points corresponding to GV (0 h), GVBD (20 h), M I (28 h) and M II (44 h) stages. The blots were immunoblotted with anti-CK2 and anti-Gapdh antibodies, respectively. **d** Protein levels of phosphorylated CK2 at specific time points corresponding to GV (0 h), GVBD (20 h), M I (28 h) and M II (44 h) stages. The blots were immunoblotted with anti-phosphoCK2 and anti-Gapdh antibodies, respectively

To examine the roles of CK2 during porcine oocyte meiotic maturation, we firstly observed its subcellular localization and protein expression patterns in different stages of meiotic maturation. Immunostaining analysis of endogenous CK2 using both CK2 and phosphorylated CK2 antibodies revealed that CK2 was present on the chromosomes from GV to MII stages and became phosphorylated after GVBD in porcine oocytes (Fig. 1a, b). Furthermore, by immunoblotting analysis, we observed that the protein expression levels of CK2 remained constant in the whole cell cycle progression of oocyte meiosis (Fig. 1c). Notably, after GVBD, the molecular weight of CK2 was apparently increased, suggesting that CK2 was phosphorylated following that developmental stage (Fig. 1c), which was further confirmed by the immunoblotting using the phosphorylated CK2 antibody (Fig. 1d).

### CK2 is essential for the meiotic progression of porcine oocytes

**Fig. 2:**
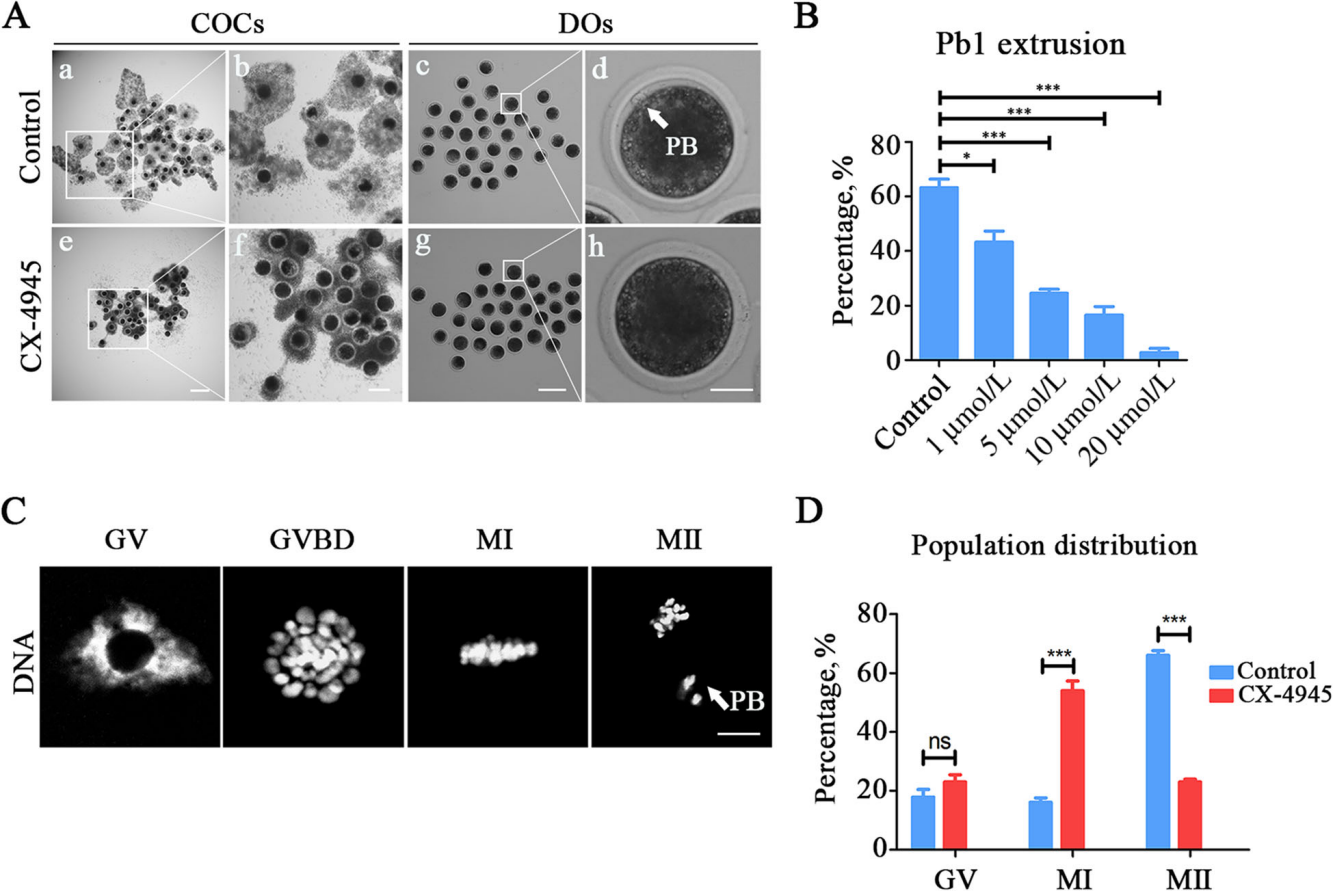
Effect of CK2 inhibition on the porcine oocyte meiotic progression. **a** Representative images of cumulus cell expansion and polar body extrusion were shown in control and CK2-inhibited oocytes cultured *in vitro* for 44 h. Oocytes were denuded following the culture to observe the polar body. COCs, cumulus-oocyte complexes; DOs, denuded oocytes. Scale bar, 500 μm (a, e); 200 μm (b, f); 250 μm (c, g); 30 μm (d, h). **b** The rate of polar body extrusion was recorded in control and different concentrations of CK2-inhibited groups (1 μmol/L, 5 μmol/L, 10 μmol/L and 20 μmol/L) after for 44 h culture. **c** Representative images of the chromosome morphology in the different developmental stages of oocyte maturation. DNA was counterstained with PI. Scale bar, 5 μmol/L. **d** The percentage of different development stages was counted in control and CK2-inhibited oocytes. Data of (**b**) and (**d**) were presented as mean percentage (mean ± SEM) of at least three independent experiments. *P < 0.05, ***P < 0.001

To investigate whether CK2 exerts a function during porcine oocyte maturation, a CK2-specific inhibitor CX-4945 was applied to block its activity. The results from Fig. 2a and b showed that treatment of oocytes with different doses of CX-4945 (1, 5, 10 and 20 μmol/L) resulted in the failure of meiotic progression by manifesting the defective expansion of cumulus cells and the reduced rate of PBE (polar body extrusion) in varying degree after 42–44 h *in vitro* culture (control: 63.2% ± 3.1%, *n* = 93; 1 μmol/L: 43.2% ± 4.1%, *n* = 90, *P* < 0.05; 5 μmol/L: 24.6% ± 1.5%, *n* = 96, *P* < 0.001; 10 μmol/L: 16.6% ± 3.1%, *n* = 99, *P* < 0.001; 20 μmol/L: 2.77% ± 1.6%, *n* = 87, *P* < 0.001; Fig. 2b). 5 μmol/L CX-4945 was applied for the subsequent analyses as this concentration has already exhibited a severe phenotype. In addition, we found that most of CK2-inhibited oocytes that did not extrude polar body were arrested at M I stage (control: 16.0% ± 1.5%, *n* = 40 vs. CX-4945: 54.0% ± 3.3%, *n* = 23, *P* < 0.001; Fig. 2c, d).

### Inhibition of CK2 leads to the activation of spindle checkpoint in porcine oocytes

**Fig. 3:**
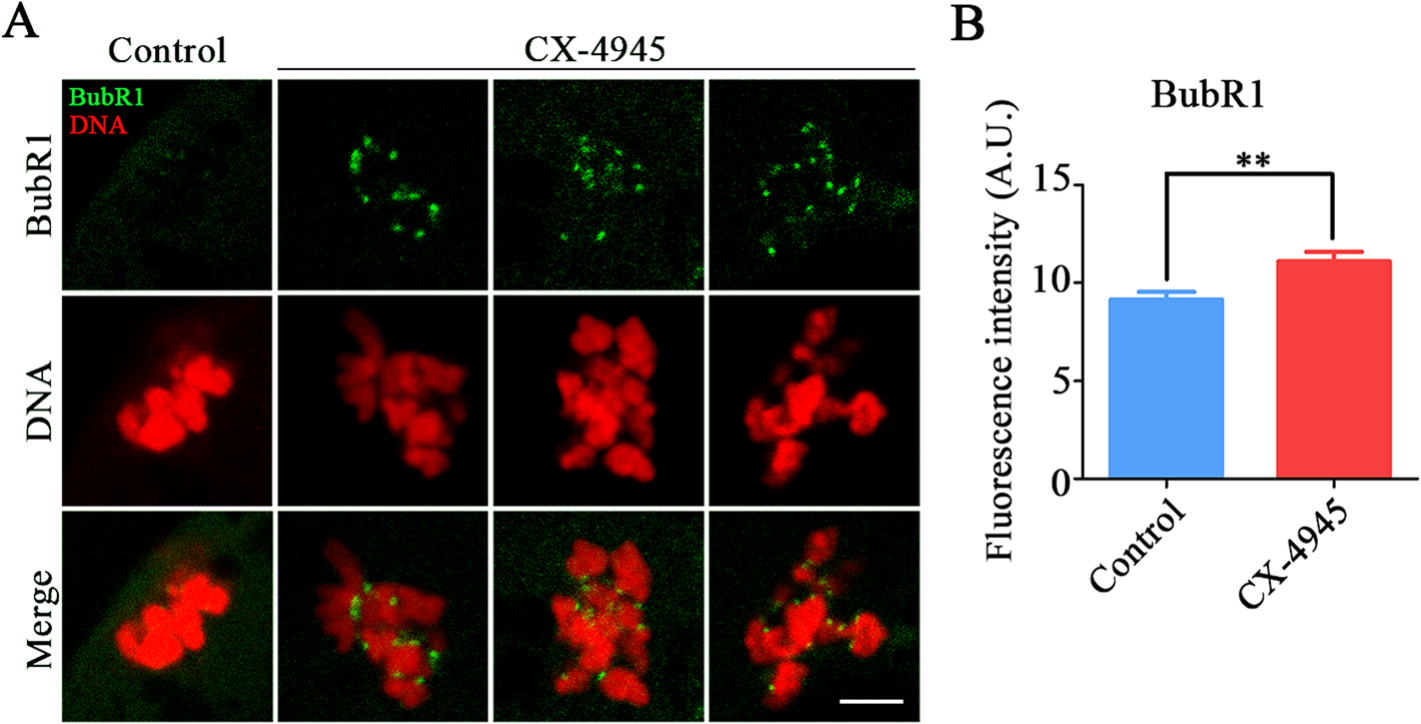
Effect of CK2 inhibition on the localization of BubR1 in porcine oocytes. **a** Localization of BubR1 at metaphase I stage in control and CK2-inhibited oocytes. Scale bar, 5 μm. **b** Quantitative analysis of the fluorescence intensity of BubR1 signals in control and CK2-inhibited oocytes. Data were presented as mean percentage (mean ± SEM) of at least three independent experiments. **P < 0.01

To ask whether M I arrest during oocyte development is caused by the activation of SAC (spindle assembly checkpoint), we stained BubR1, a core protein of SAC complexes, in CK2-inhibited oocytes. The immunofluorescence analysis showed that BubR1 was absent from chromosomes at M I stage to allow oocytes to enter the anaphase. However, when CK2 was inhibited, BubR1 still remained on the kinetochores with strong signals (Fig. 3a). Consistently, quantification of fluorescence intensity indicated that BubR1 signals were considerably higher in CK2-inhibited oocytes compared with the controls (control: 9.1 ± 0.39, *n* = 43 vs. CX-4945: 11.1 ± 0.49, *n* = 33, *P* < 0.01; Fig. 3b), implying that the arrest of CK2-inhibited oocytes at metaphase I stage is due to the activation of SAC.

### Inhibition of CK2 impairs the spindle/chromosome structure in porcine oocytes

**Fig. 4:**
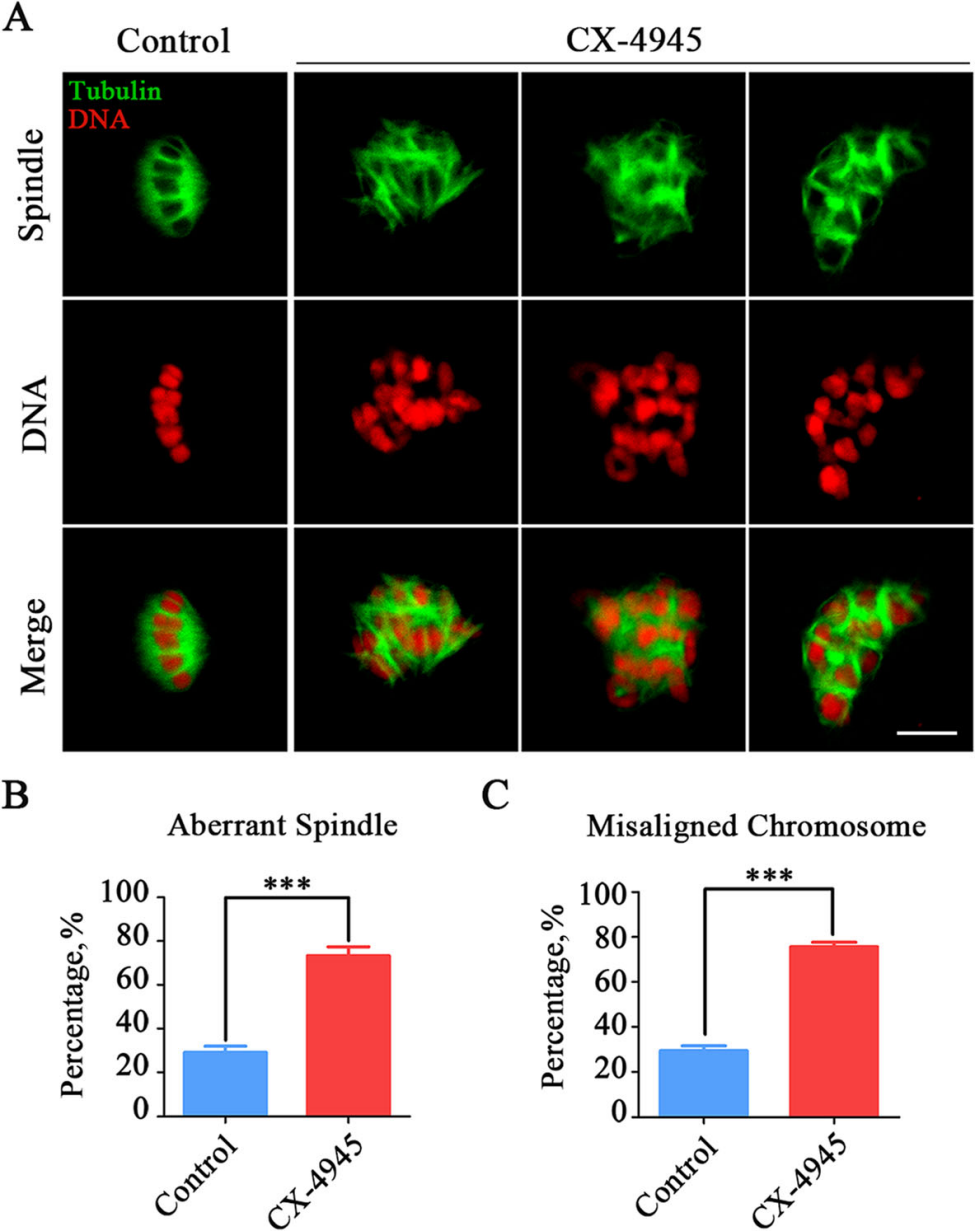
Effect of CK2 inhibition on the spindle assembly and chromosome alignment in porcine oocytes. **a** Representative images of spindle morphologies and chromosome alignment in control and CK2-inhibited oocytes. M I oocytes were immnunostained with anti-α-tubulin-FITC antibody to visualize the spindles and counterstained with PI to visualize the chromosomes. Scale bar, 5 μm. **b** The rate of aberrant spindles was recorded in control and CK2-inhibited oocytes. **c** The rate of misaligned chromosomes was recorded in control and CK2-inhibited oocytes. Data of (**b**) and (**c**) were presented as mean percentage (mean ± SEM) of at least three independent experiments. ***P < 0.001

It has been know that impairment of SAC control is always caused by the defective spindle assembly, we thus further observed the organization of spindle/chromosome structure in CK2-inhibited oocytes. To this end, spindle morphology was displayed by α-tubulin-FITC staining, and chromosome alignment was shown by PI staining. As shown in Fig. 4, the control oocytes showed a normal barrel-shaped spindle apparatus with well-aligned chromosomes at the equatorial plate (Fig. 4a). However, various disorganized spindles and misaligned chromosomes were present in CK2-inhibited oocytes (Fig. 4a). Quantitatively, the abnormal rates of spindle/chromosome structure were considerably higher in CK2-inhibited oocytes compared with the controls (control: 29.2% ± 2.8%, *n* = 99 vs. CX-4945: 73.3% ± 4.1%, *n* = 97, *P* < 0.001, spindle; control: 29.3% ± 2.2%, *n* = 96 vs. CX-4945: 75.6% ± 2.0%, *n* = 90, *P* < 0.001, chromosome; Fig. 4b, c).

### Inhibition of CK2 weakens the microtubule stability in porcine oocytes

**Fig. 5:**
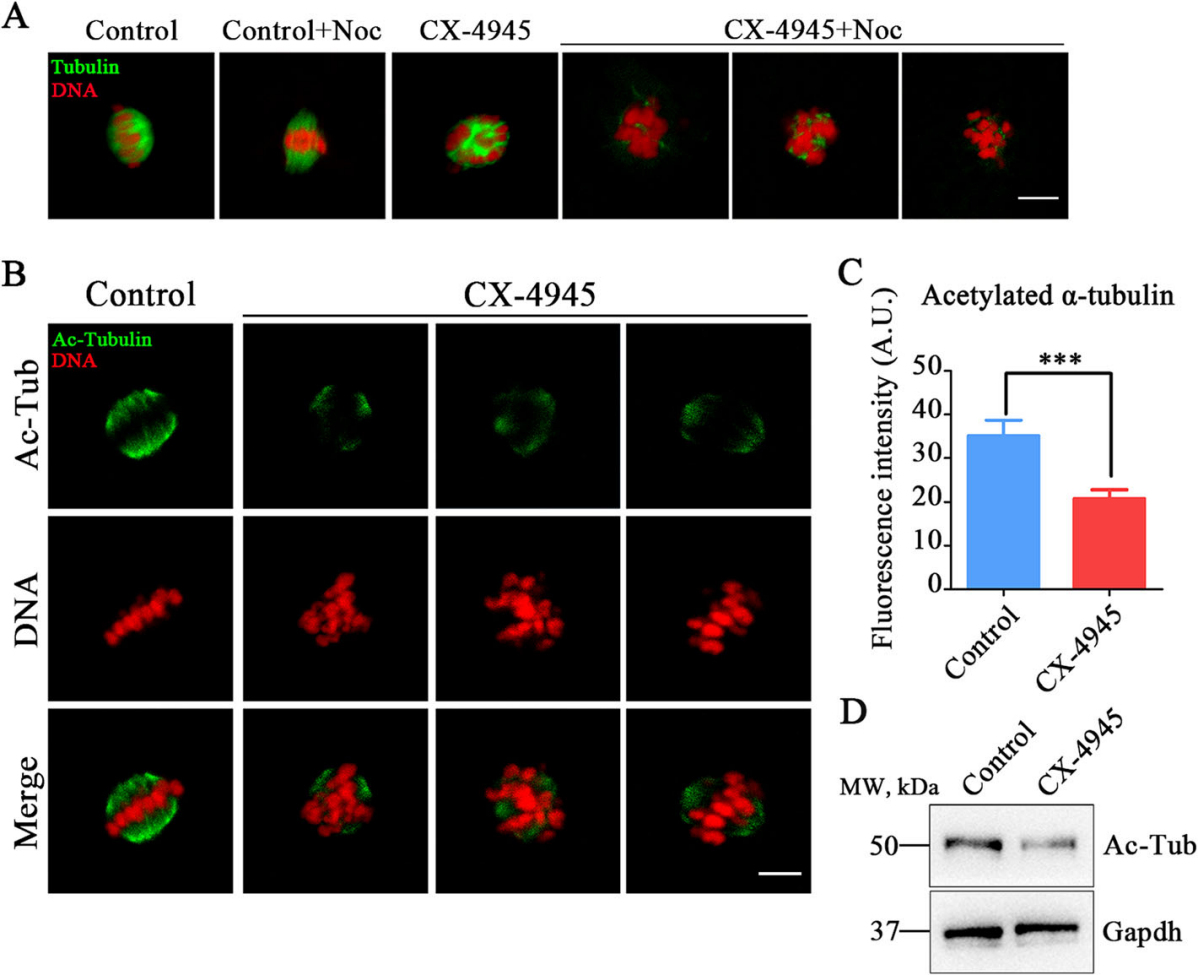
Effect of CK2 inhibition on the acetylation level of α-tubulin in porcine oocytes. **a** Representative images of microtubule fibers before and after nocodazole treatment in control and CK2-inhibited oocytes. Oocytes were immunostained with anti-α-tubulin-FITC antibody to display microtubules and counterstained with PI to visualize chromosomes. Scale bar, 10 μm. **b** Representative images of acetylated α-tubulin in control and CK2-inhibited oocytes. Oocytes were immnunostained with anti-acetyl-α-tubulin (Lys-40) antibody to assess the acetylation level of α-tubulin. Scale bar, 5 μm. **c** The fluorescence intensity of acetylated α-tubulin was measured in control and CK2-inhibited oocytes. Data were presented as mean percentage (mean ± SEM) of at least three independent experiments. ***P < 0.001. **d** The acetylation levels of α-tubulin in control and CK2-inhibited oocytes were examined by western blotting. The blots were probed with anti-acetyl-α-tubulin (Lys-40) antibody and anti-Gapdh antibody, respectively

Since the spindle assembly is controlled by the microtubule dynamics, we asked whether the aberrant spindle organization in CK2-inhibited oocytes is due to the defective microtubule stability. To test this hypothesis, we employed microtubule depolymerizing drug nocodazole to collapse microtubules. As shown in Fig. 5, in control oocytes, after nocodazole treatment for 5 min, microtubule fibers still persisted although the spindle morphologies were disrupted (Fig. 5a). However, by performing the same treatment, microtubule fibers completely disappeared in CK2-inhibited oocytes, indicative of the attenuated microtubule stability (Fig. 5a). This observation was further validated by assessing the acetylation level of α-tubulin, an index of the stabilized microtubules which has been previously documented in oocytes [23, 24]. As evaluated by the immunofluorescence analysis and the quantification of fluorescence intensity, the level of acetylated α-tubulin was remarkably decreased in CK2-inhibited oocytes as compared with the controls (control: 35.1 ± 3.5, *n* = 46 vs. CX-4945: 20.8 ± 1.9, *n* = 47, *P* < 0.001; Fig. 5b, c), consistent with the immunoblotting result (Fig. 5d). Collectively, these data indicate that inhibition of CK2 attenuates the microtubule stability, and thereby perturbing the normal spindle assembly during oocyte meiotic maturation.

### Inhibition of CK2 elevates the level of DNA damage in porcine oocytes

**Fig. 6:**
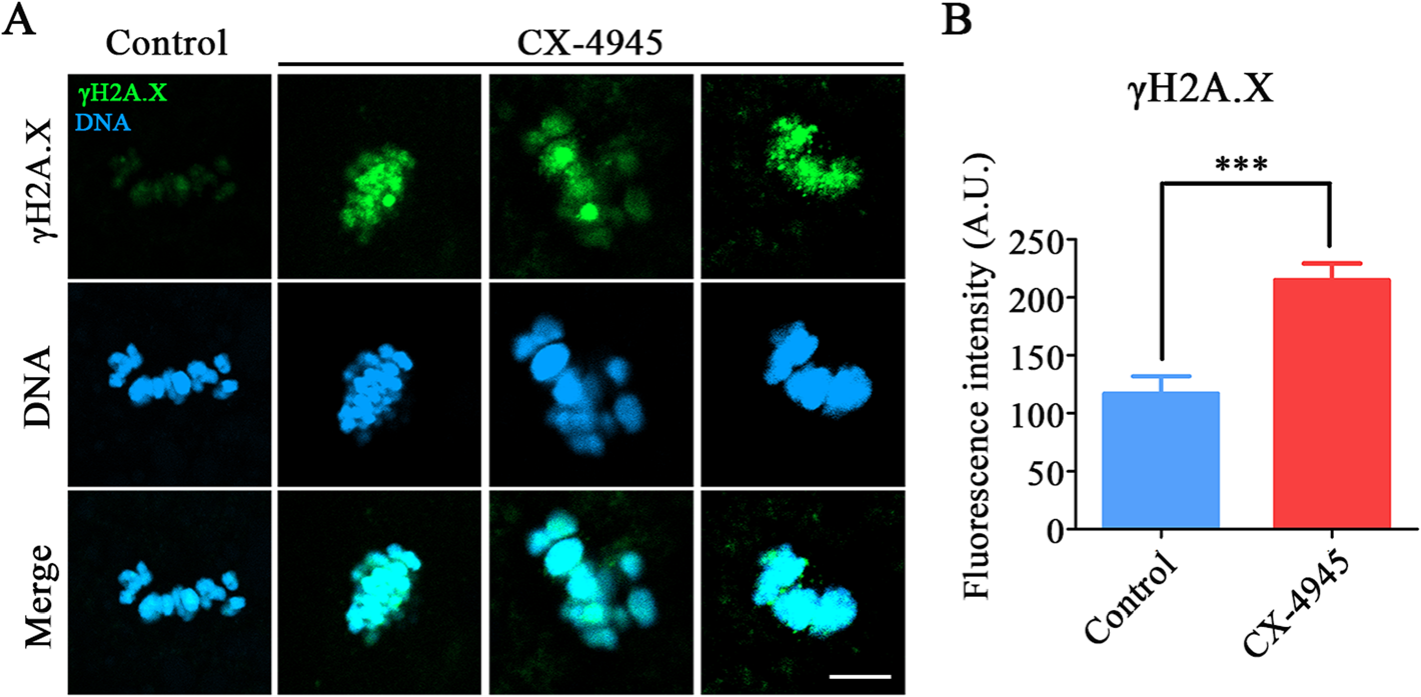
Effect of CK2 inhibition on the DNA damage level in porcine oocytes. **a** Representative images of DNA damage in control and CK2-inhibited oocytes. Oocytes were immnunostained with anti-γH2A.X antibody and counterstained with Hoechst. Scale bar, 5 μm. **b** The fluorescence intensity of γH2A.X signals was measured in control and CK2-inhibited oocytes. Data were presented as mean percentage (mean ± SEM) of at least three independent experiments. ***P < 0.001

It has been shown that CK2 locates on the DNA double-strand break sites to modulate the response to DNA damage in somatic cells [25]. Therefore, we examined whether inhibition of CK2 would affect the level of DNA damage in oocytes. To test it, we performed the immunofluorescence with γH2A.X antibody. As a result, we observed the massive accumulation of γH2A.X foci on the chromosomes in CK2-inhibited oocytes compared with the controls (control: 117 ± 14, *n* = 42 vs. CX-4945: 215 ± 13.8, *n* = 49, *P* < 0.001; Fig. 6a, b), indicative of the increased level of DNA damage.

## Discussion

CK2 is a highly conserved serine-threonine kinase implicated in multiple biological events such as spermatogenesis [18, 19], embryonic development [5, 20, 21], tumorigenesis [26] and circadian rhythms [27] etc. Inhibition of CK2 by TBB (4, 5, 6, 7-tetrabromobenzotriazole) disturbed G2/M transition in mouse zygotes and increased DNA damage level in female pronucleus [12]. On the contrary, inhibition of CK2 did not affect GVBD in mouse oocytes [12]. However, the function of CK2 in oocyte meiosis has not been well studied. Therefore, we employed porcine oocytes as a model to investigate the role of CK2 in meiosis.

Firstly we found by immunofluorescent staining that phosphorylated CK2 were localized on the chromosomes during the various developmental stages of porcine oocyte meiosis after GVBD. However, in somatic cells CK2 exhibits the ubiquitous distribution throughout the nuclear and cytoplasmic compartments [28–30]. Notably, CK2α localizes to the mitotic spindle in a phosphorylation-dependent manner and phosphorylation of CK2α facilitates binding to the peptidyl-prolyl isomerase Pin1, which is required for CK2α mitotic spindle localization [31]. The unique localization patterns in different types of cells predict their potential distinct functions. In addition, our immunoblotting result showed that CK2 was constantly expressed during the entire maturational progression of porcine oocyte meiosis and became phosphorylated after GVBD, indicating that the phosphorylated form of CK2 might be the functional molecule in porcine oocytes.

We next used a CK2-specific inhibitor, CX-4945, to examine the role of CK2 during porcine oocyte meiotic maturation. For the specificity of inhibitor CX-4945, in cell-based functional assays, CX-4945 treatment at 10 μmol/L is inactive against its potential other targets FLT3, PIM1, and CDK1 (Selleck website). In our current study, we used 5 μmol/L CX-4945 for the experiments, which probably has little effect on the activity of FLT3, PIM1, and CDK1. Our data showed that inhibition of CK2 activity impaired the oocyte meiotic progression by displaying the dramatically reduced frequency of PBE, indicating that CK2 is required for the normal maturation of porcine oocytes. Because the activity of CK2 in the cumulus cells was also blocked, the maturation failure of oocytes might partially result from the poor expansion of cumulus cells. Further analysis of arrested oocytes by DNA staining revealed that the meiotic failure of most CK2-inhibited oocytes occurred at metaphase I stage, suggesting that SAC might be activated to inhibit the onset of anaphase. This assumption was then verified by the observation that BubR1, a core component of SAC proteins, was still present at kinetochores in M I CK2-inhibited oocytes.

To explore the cause leading to SAC activation at M I stage, we examined the spindle/chromosome structure which has been previously shown to be involved in the SAC control in oocytes [32]. As expected, we observed that inhibition of CK2 caused a significant increase in the abnormalities of spindle morphology and chromosome alignment in porcine oocytes, indicating that compromised spindle assembly induced the SAC activation at M I stage to block metaphase-anaphase transition and polar body extrusion upon CK2 inhibition.

The abnormal spindle organization prompted us to further evaluate the dynamics of microtubules. Both nocodazole treatment and the level of acetylated α-tubulin validated that microtubule stability was weakened in CK2-inhibited oocytes, implying that defective microtubule dynamics disrupted spindle assembly, thereby activating SAC to impede the normal meiotic progression of porcine oocytes.

Another critical factor that would induce the M I arrest of oocyte meiosis is the occurrence of DNA damage [33]. Previous studies have revealed that CK2 takes critical parts in the DNA damage response and repair, we thus assessed the DNA damage level in CK2-inhibtied oocytes. Our findings verified our hypothesis that inhibition of CK2 caused a prominent increase in the accumulation of DNA damage, which might be another critical cause resulting in the meiotic defects of porcine oocytes.

## Conclusions

Taken together, our findings provide evidence documenting that CK2 is essential for the meiotic progression of porcine oocytes by regulating spindle assembly and DNA damage repair. Inhibition of CK2 compromises these two events, thereby activating SAC and resulting in meiotic arrest at the M I stage.

## Data Availability

All data generated or analyzed during this study are available from the corresponding author on reasonable request.

## Abbreviations

CK2: Casein kinase 2
COCs: Cumulus-oocyte complexes
DOs: Denuded oocytes
EGF: Epidermal growth factor
FSH: Follicle-stimulating hormone
LH: Luteinizing hormone
PFA: Paraformaldehyde
PI: Propidium iodide
SAC: Spindle assembly checkpoint

## References

[1] Litchfield DW (2003). Protein kinase CK2: structure, regulation and role in cellular decisions of life and death. Biochem J.

[2] Guerra B, Issinger OG, Wang JY (2003). Modulation of human checkpoint kinase Chk1 by the regulatory beta-subunit of protein kinase CK2. Oncogene..

[3] Yde CW, Olsen BB, Meek D, Watanabe N, Guerra B (2008). The regulatory beta-subunit of protein kinase CK2 regulates cell-cycle progression at the onset of mitosis. Oncogene..

[4] Ahmad KA, Wang G, Unger G, Slaton J, Ahmed K (2008). Protein kinase CK2--a key suppressor of apoptosis. Adv Enzym Regul.

[5] Lin F, Cao SB, Ma XS, Sun HX (2017). Inhibition of casein kinase 2 blocks G2/M transition in early embryo mitosis but not in oocyte meiosis in mouse. J Reprod Dev.

[6] Loizou JI, El-Khamisy SF, Zlatanou A, Moore DJ, Chan DW, Qin J (2004). The protein kinase CK2 facilitates repair of chromosomal DNA single-strand breaks. Cell..

[7] Ghavidel A, Schultz MC (2001). TATA binding protein-associated CK2 transduces DNA damage signals to the RNA polymerase III transcriptional machinery. Cell..

[8] Dixit D, Sharma V, Ghosh S, Mehta VS, Sen E (2012). Inhibition of casein kinase-2 induces p53-dependent cell cycle arrest and sensitizes glioblastoma cells to tumor necrosis factor (TNFalpha)-induced apoptosis through SIRT1 inhibition. Cell Death Dis.

[9] Zhao T, Jia H, Li L, Zhang G, Zhao M, Cheng Q (2013). Inhibition of CK2 enhances UV-triggered apoptotic cell death in lung cancer cell lines. Oncol Rep.

[10] Lebrin F, Chambaz EM, Bianchini L (2001). A role for protein kinase CK2 in cell proliferation: evidence using a kinase-inactive mutant of CK2 catalytic subunit alpha. Oncogene..

[11] Homma MK, Wada I, Suzuki T, Yamaki J, Krebs EG (2005). CK2 phosphorylation of eukaryotic translation initiation factor 5 potentiates cell cycle progression. Proc Natl Acad Sci U S A.

[12] Liang QX, Wang ZB, Lin F, Zhang CH, Sun HM, Zhou L, et al. Ablation of beta subunit of protein kinase CK2 in mouse oocytes causes follicle atresia and premature ovarian failure. Cell Death Dis. 2018;9(5):508. 10.1038/s41419-018-0505-1 PMC5938699 29725001

[13] So KS, Rho JK, Choi YJ, Kim SY, Choi CM, Chun YJ (2015). AKT/mTOR down-regulation by CX-4945, a CK2 inhibitor, promotes apoptosis in chemorefractory non-small cell lung cancer cells. Anticancer Res.

[14] Bliesath J, Huser N, Omori M, Bunag D, Proffitt C, Streiner N (2012). Combined inhibition of EGFR and CK2 augments the attenuation of PI3K-Akt-mTOR signaling and the killing of cancer cells. Cancer Lett.

[15] Kim HM, Jeong I, Kim HJ, Kang SK, Kwon WS, Kim TS (2018). Casein kinase 2 inhibitor, CX-4945, as a potential targeted anticancer agent in gastric Cancer. Anticancer Res.

[16] Rabalski AJ, Gyenis L, Litchfield DW (2016). Molecular pathways: emergence of protein kinase CK2 (CSNK2) as a potential target to inhibit survival and DNA damage response and repair pathways in Cancer cells. Clin Cancer Res.

[17] Kim J, Kim SH (2012). Druggability of the CK2 inhibitor CX-4945 as an anticancer drug and beyond. Arch Pharm Res.

[18] Xu X, Toselli PA, Russell LD, Seldin DC (1999). Globozoospermia in mice lacking the casein kinase II alpha' catalytic subunit. Nat Genet.

[19] Escalier D, Silvius D, Xu X (2003). Spermatogenesis of mice lacking CK2alpha': failure of germ cell survival and characteristic modifications of the spermatid nucleus. Mol Reprod Dev.

[20] Lou DY, Dominguez I, Toselli P, Landesman-Bollag E, O'Brien C, Seldin DC (2008). The alpha catalytic subunit of protein kinase CK2 is required for mouse embryonic development. Mol Cell Biol.

[21] Buchou T, Vernet M, Blond O, Jensen HH, Pointu H, Olsen BB (2003). Disruption of the regulatory beta subunit of protein kinase CK2 in mice leads to a cell-autonomous defect and early embryonic lethality. Mol Cell Biol.

[22] Lieberman SL, Ruderman JV (2004). CK2 beta, which inhibits Mos function, binds to a discrete domain in the N-terminus of Mos. Dev Biol.

[23] Xie Y, Cheng M, Lu S, Yuan Q, Yang D, Chen Y (2018). Eg5 orchestrates porcine oocyte maturational progression by maintaining meiotic organelle arrangement. Cell Div.

[24] Tang F, Pan MH, Wan X, Lu Y, Zhang Y, Sun SC (2018). Kif18a regulates Sirt2-mediated tubulin acetylation for spindle organization during mouse oocyte meiosis. Cell Div.

[25] Olsen BB, Wang SY, Svenstrup TH, Chen BP, Guerra B (2012). Protein kinase CK2 localizes to sites of DNA double-strand break regulating the cellular response to DNA damage. BMC Mol Biol.

[26] Maslyk M, Janeczko M, Martyna A, Kubinski K (2017). CX-4945: the protein kinase CK2 inhibitor and anti-cancer drug shows anti-fungal activity. Mol Cell Biochem.

[27] Allada R, Meissner RA (2005). Casein kinase 2, circadian clocks, and the flight from mutagenic light. Mol Cell Biochem.

[28] Laramas M, Pasquier D, Filhol O, Ringeisen F, Descotes JL, Cochet C (2007). Nuclear localization of protein kinase CK2 catalytic subunit (CK2alpha) is associated with poor prognostic factors in human prostate cancer. Eur J Cancer.

[29] Faust RA, Niehans G, Gapany M, Hoistad D, Knapp D, Cherwitz D (1999). Subcellular immunolocalization of protein kinase CK2 in normal and carcinoma cells. Int J Biochem Cell Biol.

[30] Faust RA, Niehans GA, Gapany M, Adams GL, Ahmed K (1995). Subcellular immunolocalization of protein kinase CK2 in squamous cell carcinomas of the head and neck: correlation with CK2 activity. Otolaryngol Head Neck Surg.

[31] St-Denis NA, Bailey ML, Parker EL, Vilk G, Litchfield DW (2011). Localization of phosphorylated CK2alpha to the mitotic spindle requires the peptidyl-prolyl isomerase Pin1. J Cell Sci.

[32] Radonova Lenka, Svobodova Tereza, Skultety Michal, Mrkva Ondrej, Libichova Lenka, Stein Paula, Anger Martin (2019). ProTAME Arrest in Mammalian Oocytes and Embryos Does Not Require Spindle Assembly Checkpoint Activity. International Journal of Molecular Sciences.

[33] Lane SIR, Morgan SL, Wu T, Collins JK, Merriman JA, ElInati E (2017). DNA damage induces a kinetochore-based ATM/ATR-independent SAC arrest unique to the first meiotic division in mouse oocytes. Development.

